# Bacteriostatic and Immunomodulatory Effect of Xylocaine in the Context of *in Vitro* Bladder Epithelial Cell Infection

**DOI:** 10.1021/acsbiomedchemau.4c00070

**Published:** 2024-12-16

**Authors:** John Kerr White, Yundi Yin, Soumitra Mohanty, Natalia Ferraz, Annelie Brauner

**Affiliations:** † Department of Microbiology, Tumor and Cell Biology, Karolinska Institutet, 17177 Stockholm, Sweden; ‡ Division of Clinical Microbiology, Karolinska University Hospital, 17176 Stockholm, Sweden; § Nanotechnology and Functional Materials, Department of Materials Science and Engineering, Uppsala University, Box 35, 75103 Uppsala, Sweden

**Keywords:** analgesic agents, immunomodulation, *E. coli*, UTI, xylocaine

## Abstract

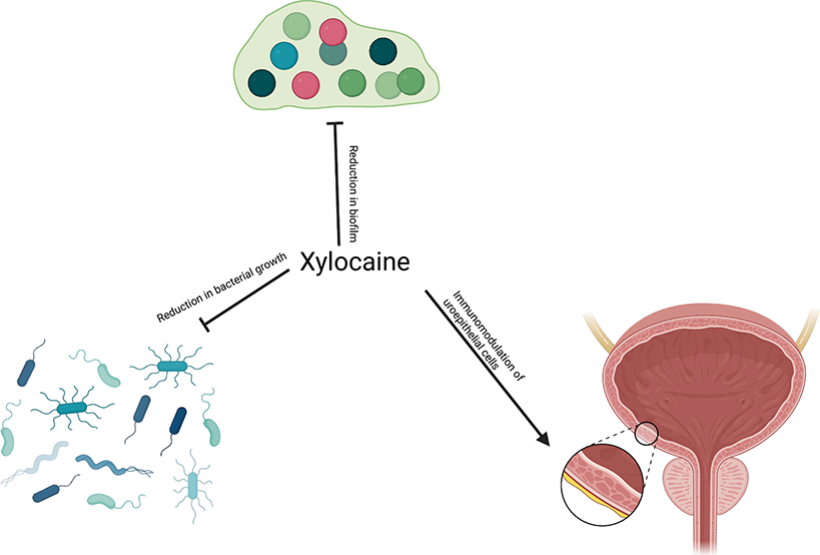

The application of urinary catheters is associated with pain and discomfort. Several topical medications are available to ease catheter insertion, including xylocaine. Here we report that xylocaine, although not classified as an antibacterial agent, has bacteriostatic properties against both Gram-positive and Gram-negative etiological agents of urinary tract infections (UTIs). Xylocaine reduces the amount of biofilm formed by ESBL- and non-ESBL-producing *E. coli*. In addition, xylocaine possesses slight immunomodulatory properties in uroepithelial cells, and treatment of uroepithelial cells prior to infection reduces bacterial loads in the supernatant. In conclusion, xylocaine has multifaceted positive effects when used during the insertion of urinary catheters.

Catheter associated urinary tract infections (CAUTIs), both after short- and long-term use, is one of the most common medical-device-associated infections worldwide.^1^ In particular, patients with long-term urinary catheters are colonized by bacteria within weeks of implantation and run increased risk of developing recurrent UTIs, and even life-threatening urosepticaemia.

Pain and discomfort are common symptoms associated with the application of urinary catheters. To reduce this, topical numbing agents, including xylocaine, are frequently used. Earlier research has suggested modest antibacterial properties of xylocaine and other anesthetic agents,^2,3^ but the mechanism has been poorly understood. Considering the frequent use of both short- and long-term residence of urinary catheters within the urinary tract, we evaluated the possible immunomodulatory effect of xylocaine on resident uroepithelial cells, in addition to its potential antibacterial properties.

Given the plethora of bacterial species capable of causing CAUTI in healthcare settings, we challenged a variety of common bacterial agents known to cause UTIs. Interestingly, we demonstrate that xylocaine has growth-inhibitory properties to both Gram-positive and Gram-negative bacteria including those which are antibiotic sensitive, extended spectrum beta-lactamase (ESBL) producing, and multidrug resistant (Figures 1A–F).

**Figure 1:**
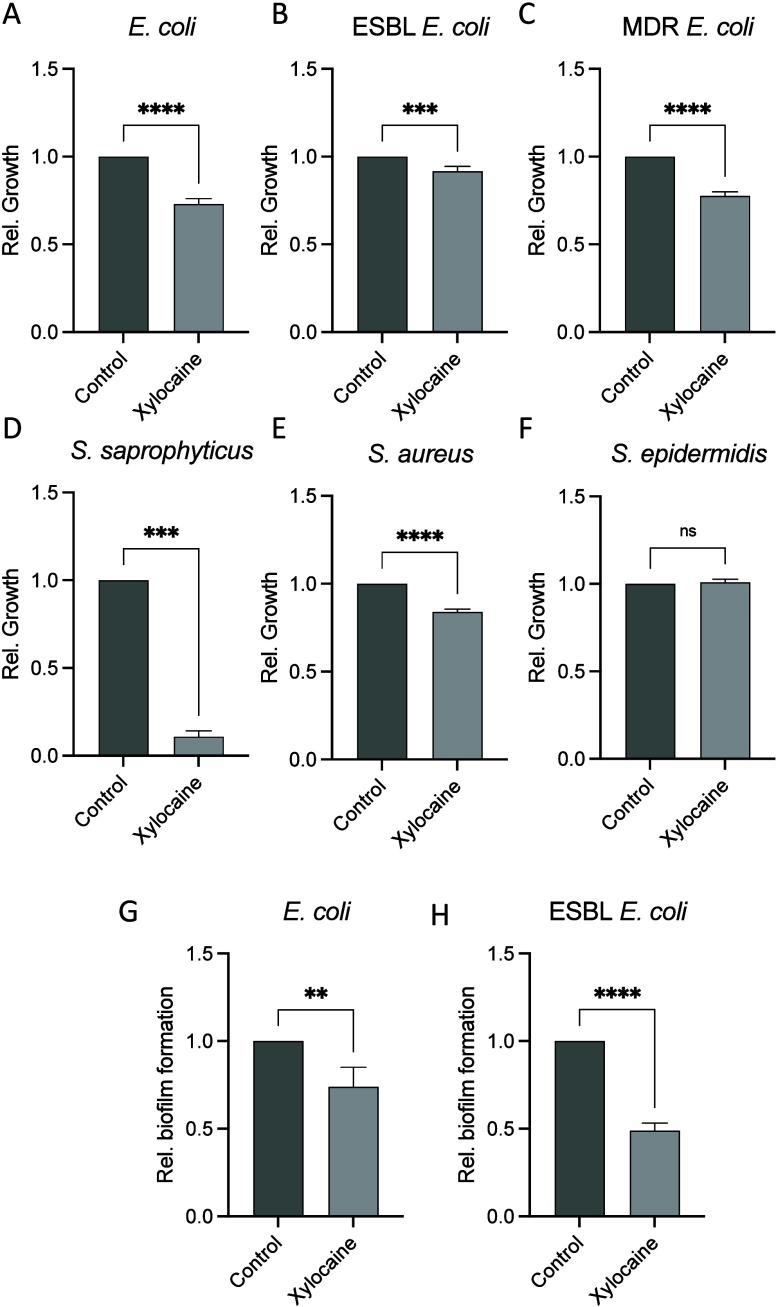
Antimicrobial activity of xylocaine against Gram-positive and Gram-negative bacteria. Relative growth of clinical isolates of *E. coli* (A), ESBL producing *E. coli* (B), MDR *E. coli* (C), *S. saprophyticus* (D), *S. aureus* (E), and *S. epidermidis* (F) when challenged with 4 mM xylocaine relative to vehicle control. Reduction in biofilm formed by *E. coli* CFT073 (G) and ESBL producing *E. coli* CCUG 58543 (H) when treating with xylocaine versus vehicle control. All *in vitro* experiments were performed in triplicate with 3 independent experiments. Statistical outliers, defined by Grubb’s test, were excluded. Statistics was performed using unpaired Student’s *t* test or Mann–Whitney as appropriate. **p < 0.01, ***p < 0.001, and ****p < 0.0001.

During CAUTIs, uropathogens utilize diverse strategies to evade eradication by host immune responses. These include extracellular compounds involved in surface adhesion and the secretion of exopolysaccharides to form a robust matrix. In combination, this forms a biofilm able to shield from bacterial eradication strategies. The formation of biofilm begins shortly after initial adherence to a surface.^4^ Despite a mostly low level of biofilm production, we here observed a significant reduction in the amount of new biofilm produced by antibiotic sensitive and resistant clinical isolates of *E. coli* after 72 h of culture in the presence of xylocaine (Figures 1G and H).

During infection, the first line of defense initiated by resident epithelial and immune cells is by releasing endogenous antimicrobial peptides (AMPs) as well as reactive oxygen and nitrogen species. This constitutes a part of the body’s weaponry for preventing and combating UTIs, and disruptions to this prevent a self-resolved infection.^5−7^ In uroepithelial cells, xylocaine treatment led to a higher release of free nitric oxide, with a slight but significant reduction in the release of free reactive oxygen species (Figures 2A and B). Conditioned media taken from uroepithelial cells treated with xylocaine induced a decrease in the microbial burden (Figure 2C). Together these findings suggest that xylocaine most likely also has beneficial, presumably indirect, effects in reducing the microbial burden during initial infection by creating a more hostile environment to invading bacteria.

**Figure 2:**
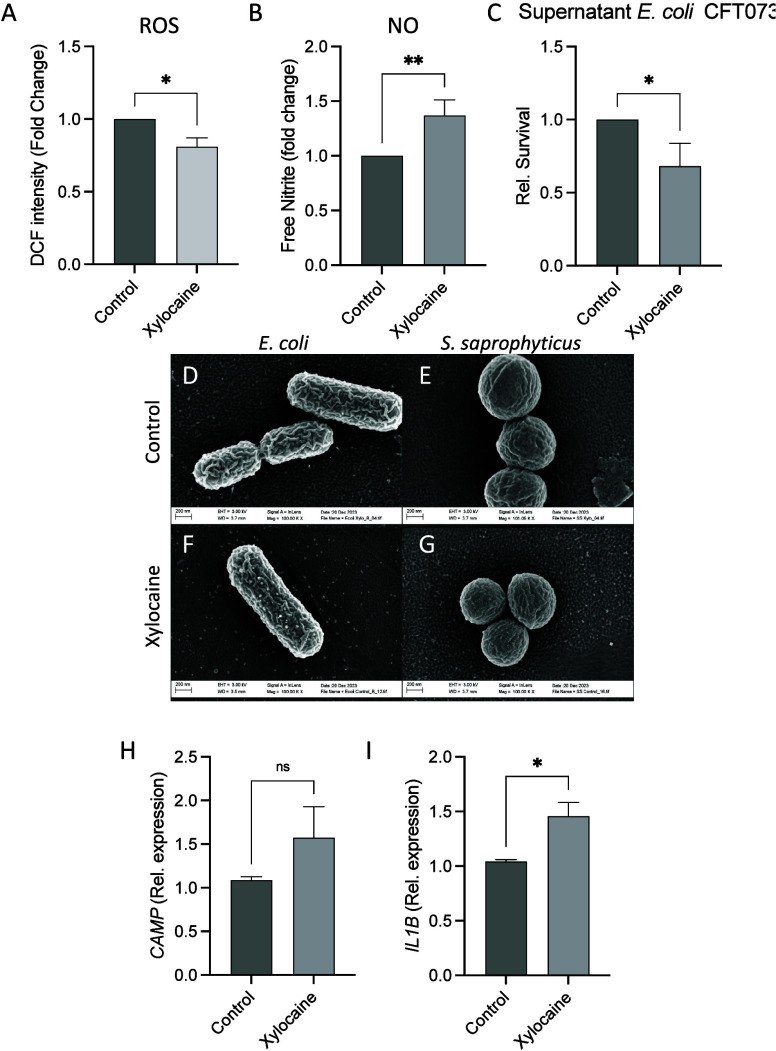
Indirect and immunomodulatory effect of xylocaine on uroepithelial cells. Uroepithelial cells treated with xylocaine had a reduction in the release of free radical oxygen species (ROS; Figure 2A) but an increase in free nitric oxide (NO; Figure 2B). Conditioned media taken from cells treated with xylocaine displayed a reduction in bacterial load as compared with conditioned media from cells treated with vehicle control (Figure 2C). Representative scanning electron microscopy images of *E. coli* CFT073 (Figures 2D and F) and *S. saprophyticus* ATCC 15305 (Figures 2E and G) with or without treatment with xylocaine. mRNA expression of *CAMP* and *IL1B* in uroepithelial cells after treatment with xylocaine revealed a nonsignificant increase in *CAMP* but a significant increase in *IL1B* (Figures 2H and I). *In vitro* experiments (Figures 2A–C and Figures 2H and I) were performed in triplicate with 3 independent experiments. Statistical outliers, defined by Grubb’s test, were excluded. Statistics was performed using unpaired Student’s *t* test or Mann–Whitney as appropriate. *p < 0.05 and **p < 0.01.

Cationic compounds are known to act directly on the bacterial membrane to cause cell lysis.^8^ Given that xylocaine is a cationic compound and possesses moderate antibacterial properties, we utilized scanning electron microscopy (SEM) to visualize the effect of xylocaine on bacterial morphology. Interestingly, we observed no clear morphological changes between xylocaine-treated and control cells (Figures 2D–G), thereby suggesting that its antibacterial effect is not mediated by bacterial membrane damage, in the xylocaine concentrations used.

It is well-known that uroepithelial cells produce AMPs and interleukins upon stimulation by metabolites and during infection.^9,10^ However, AMP expression and release can also be triggered by synthetic cationic compounds, which prime the innate immune response to bacterial attack.^8^ Therefore, we assessed whether xylocaine would initiate an immunomodulatory response in uroepithelial cells.

Stimulation of the human uroepithelial cell line 5637 with xylocaine significantly increased the expression of *IL1B.* However, we did not see a corresponding upregulation on either the mRNA or protein level for the expression of AMP LL-37 (Figures 2H and I). This could be due to differential regulation in the gene expression and the time point used in the analysis. The lack of increased LL-37 in uninfected epithelial cells is not a detriment, as excess LL-37 is associated with a self-sustaining inflammatory loop^11^ and can induce local epithelial cell death.^12^

Overall, we demonstrate that xylocaine has antibacterial properties against etiological agents of CAUTIs, including those that are drug resistant. The increased innate immune response seen in stimulated uroepithelial cells would help prevent the adherence of bacteria to the catheter and reduce the risk of CAUTI. The versatility of the antibacterial effect of xylocaine against both Gram-positive and Gram-negative pathogens in concentrations far below cellular cytotoxicity is noteworthy. In addition, the supernatant from stimulated uroepithelial cells treated with xylocaine displayed a reduction in the bacterial load. As a commercially available analgesic, xylocaine has beneficial properties beyond reducing pain, especially for patients with short-term catheterization. Despite the bacteriostatic and immunomodulatory effects demonstrated, a few limitations warrant considerations. In the present study, investigating more uropathogenic bacteria and using more than one cell line would have increased our understanding of the xylocaine effect. Further, higher xylocaine concentrations, better mimicking the clinical use, might have an increased antibacterial effect. However, as the commercial formulations are in gel form, it was for us impossible to pipet. Using a dissolved powder form of xylocaine, the highest possible concentration was still much lower than that of the gel. To conclude, we here demonstrate that xylocaine has a multifaceted and beneficial repertoire beyond easing pain.

## Methods

### Xylocaine Stock

Xylocain (Sigma-Aldrich) was dissolved in 99.8% EtOH to a final concentration of 100 mM.

### Bacteria

The following bacterial type strains were used: *Escherichia coli* CFT073, ESBL-producing *E. coli* (CCUG 58543), *Staphylococcus aureus* (ATCC 29213), *S. epidermidis* (ATCC 12228), and *S. saprophyticus* (ATCC 15305). Clinical isolates, including all MDR *E. coli* isolates, were obtained from the Department of Clinical Microbiology, Karolinska University Hospital, Solna, Sweden. All clinical isolates were species identified using biochemical typing and MALDI-TOF MS. ESBL and MDR *E*. *coli* isolates had their resistance patterns confirmed by routine clinical diagnostic parameters.

All bacteria were cultured aerobically overnight on blood agar at 37 °C.

### Bacterial Inhibition Assay

The effect of 4 mM xylocaine was evaluated against the type strains and the 10 clinical bacterial isolates of each species by adding 50 μL of 10^6^ CFU/mL to 150 μL Mueller Hinton Broth that contained a final concentration of 4 mM xylocaine and incubated overnight at 37 °C. A vehicle control of ethanol was used as the control. The results were read spectrophotometrically at 595 nm. Relative growth was evaluated and normalized to controls.

### Biofilm Inhibition

To investigate if xylocaine can prevent the formation of bacterial biofilm, the crystal violet assay was used with methods as described previously, exposing the bacteria to 4 mM xylocaine.^4,13^ A vehicle control of ethanol was used as the control. Relative growth was evaluated and normalized to controls.

### Scanning Electron Microscopy

Scanning electron microscopy (SEM) was used to evaluate bacterial morphology after treatment with xylocaine or a vehicle control. SEM was performed using the method as previously described.^4^

### Cell Line and Culture Conditions

Human uroepithelial cells 5637 (HTB-9, American Type Culture Collection) were cultured in RPMI 1640 and supplemented with 10% heat inactivated fetal bovine serum (FBS; Life Technologies). All cells were incubated at 37 °C with 5% CO_2_ and 80% humidity. 5637 cells were seeded for ∼80% confluency in 24-well plates. Cells were treated with 4 mM xylocaine or ethanol control for 30 min prior to harvesting the supernatant.

### Conditioned Media Experiments

Harvested cell free supernatant from cells treated with or without xylocaine is hereby referred to as conditioned media. 50 μL of 10^6^ CFU/mL *E. coli* CFT073 was added to 500 μL of conditioned media and incubated at 37 °C for 30 min, after which viable count was performed on blood agar. Relative growth was evaluated and normalized to controls.

### Cell Infection Assays

Cell experiments were carried out in 24-well cell culture plates. Cells were grown in the presence of 4 mM xylocaine for 24 h prior to the infection. After 24 h, old media was removed and replaced with fresh media supplemented with 4 mM xylocaine, without antibiotics and serum. 10^6^ CFU/mL (MOI 5) *E. coli* CFT073 was added to 80% confluent pretreated cells and incubated in 37 °C at 5% CO_2_ and 80% humidity. After 30 min, the cells were washed once with PBS and harvested for further analysis. Survival was evaluated and normalized to untreated controls.

### RNA Extraction and Real-Time PCR Analysis

RNA extraction, cDNA synthesis, and quantitative PCR (qPCR) were performed as previously described.^3^ Gene targets in this study included the following: *ACTB* (Fw: AAG AGA GGC ATC CTC ACC CT, Rv: TAC ATC GCT GGG GTG TTG), *IL1B* (Fw: CAC GAT GCA CCT GTA CGA TCA, Rv: GTT GCT CCA TAT CCT GTC CCT), and *CAMP* (Fw: ACC CAG CAG GGC AAA TCT, Rv: GAA GGA CGG GCT GGT GAA). Gene expression was evaluated and normalized to the housekeeping gene (*ACTB*).

### Free Radical Formation Assay

5637 cells were treated with 4 mM xylocaine for 24 h prior to infection with MOI 5 *E. coli* CFT073 for 30 min. Supernatants were collected and mixed with equal volumes of Griess reagent (Invitrogen) based on the manufacturer’s protocol. Optical density was measured at 550 nm, and free nitrite was evaluated and normalized to EtOH treated control cells. For total ROS analysis, 10 μM DCFH-DA (Sigma) was added to the cells, and the cells were incubated at 37 °C and 5% CO_2_ for another 2 h. Fluorescence intensity was measured at excitation 485 nm and emission 530 nm (Fluostar Omega). Relative growth was evaluated and normalized to controls.

### Statistical Methods

All statistical tests were performed in GraphPad Prism version 9. For in vitro analysis using human uroepithelial cells, statistical outliers defined by Grubb’s test were excluded. Data were obtained from Student’s unpaired *t* test or Mann–Whitney as appropriate.
